# Integrated analysis of muscle transcriptome, miRNA, and proteome of Chinese indigenous breed Ningxiang pig in three developmental stages

**DOI:** 10.3389/fgene.2024.1393834

**Published:** 2024-05-14

**Authors:** Wenwu Chen, Yu Xiao, Fang Yang, Sui Liufu, Yan Gong, Zhi Li, Shuo Zhang, Shengguo Tang, Biao Li, Haiming Ma

**Affiliations:** ^1^ College of Animal Science and Technology, Hunan, Agricultural University, Changsha, Hunan, China; ^2^ Yunnan Southwest Agriculture and Animal Husbandry Group Co., Ltd., Kunming, Yunnan, China; ^3^ Institute of Yunnan Circular Agricultural Industry, Puer, Yunnan, China; ^4^ College of Animal and Veterinary Sciences, Southwest Minzu University, Chengdu, China

**Keywords:** Ningxiang pig, longissimus dorsi muscle, transcriptome, proteome, IMF

## Abstract

The Ningxiang pig, a distinguished local breed in China, is recognized for its good meat quality traits. This study examines the proteomics of Ningxiang pigs at three developmental stages and delves into the upstream transcriptomics of these proteomics. Such an analysis facilitates a deeper understanding of the molecular interplay between proteins and transcriptomes in the Ningxiang pig muscle, influencing muscle growth and development. In this research, we analyzed the muscles of Ningxiang pigs at three developmental stages: 30 days in weaned piglets, 90 days in nursery pigs, and 210 days in late fattening pigs. There a total of 16 differentially co-expressed miRNAs (ssc-miRNA-1, ssc-miRNA-378, ssc-miRNA-143, ssc-miRNA-30e, etc.), 74 differentially co-expressed mRNA (*PLIN3*, *CPT2*, *IGF2* and *HSP90AB1*, etc.) have been identified in the three stages. 572 differentially abundant proteins (DAPs) (*APOC3*, *NDUFA2*, *HSPD1*, *ATP5E*, *PDHA1*, etc.) were readily identified by comparing different time periods. According to the KEGG enrich pathway results that DAPs most enriched in growth and development pathways, immune mechanism pathways and maintaining functions of physical. Through short time-series expression miner (STEM) association analysis, a total of 571 negative miRNA-mRNA interaction pairs and 2 negative miRNA-mRNA-protein (Chr05_11955-Pig.17268.1-*ATP5F1B*, ssc-miR-194a-3p-Pig.15802.1-*ACY1*) interaction pairs were found. Our study provides a theoretical basis on molecular mechanism for the study of IMF deposition, muscle growth and immunity in Ningxiang pig breed.

## 1 Introduction

As the global human population continues to rise, there will be increasing pressure on resources, leading to a heightened demand for meat products, including pork. Regardless of the influence of fatty acid added in feed, current commercial pigs, characterized by a higher percentage of saturated fatty acids (SFA) and a lower content of polyunsaturated fatty acids (PUFA) and lipid-soluble antioxidant vitamins, therefore some scholars have focused on local breeds of pigs with enhanced nutritional and flavor profiles. The Ningxiang pig, a distinctive fat-type local breed in China, is highly esteemed by consumers for its elevated intramuscular fat (IMF) content, palatable meat, and unique flavor (Feng et al., 2012). Research indicates that the IMF in Ningxiang pigs is approximately 5%, significantly surpassing that of commercial pigs, which stands at 2% (He et al., 2020). However, Ningxiang pigs have an extended fattening period compared to commercial breeds like the Duroc, leading to increased feeding costs. The number of a pig’s muscle fibers is predetermined post-birth, and the equilibrium between protein synthesis and degradation in these fibers, alongside the muscle fat deposition rate, significantly influences the pig’s growth and developmental pace (Rudar et al., 2019). Thus, a deeper exploration of muscle growth is warranted.

Existing studies on muscle growth, development, and IMF predominantly derive from transcriptome and genome analyses (Cai et al., 2018; Igata et al., 2019; Zhao et al., 2019; Albuquerque et al., 2020). For instance, key muscle development regulators include myogenic regulatory factors (*MRFs*) (Aase-Remedios et al., 2020), myocyte enhancer factor 2 (*MEF2*) (Taylor and Hughes, 2017) and Myostatin (*MSTN*) (Dankbar et al., 2015); *PPARG* (Huang et al., 2020), *PLIN1* (Li et al., 2020), *AQP3* (Mi et al., 2018), *MYH3* (Bruderer et al., 2015), and *MTCH2* (Hou et al., 2020) have been identified as pivotal for IMF deposition. However, the transcriptome’s constraints make it challenging to align many mRNA transcription levels with proteome data, often leading to discrepancies between transcriptome outcomes and actual proteome findings (Schwanhäusser et al., 2011). The expression level of mRNA was detected with high or significant differences, but different results were found when detecting the corresponding protein abundance. Resulting in the expression of mRNA measured by experiment is not consistent with the real protein situation (Ai et al., 2023; Wang et al., 2023; Yu et al., 2023). Existing literature also underscores the influence of age on gene expression levels in mammalian tissues (Ahn et al., 2019; Bloom et al., 2020). Notably, there’s a research gap concerning the proteomics and transcriptomics of Ningxiang pigs’ longissimus dorsi at varied developmental stages.

In conclusion, proteomics significantly influence muscle growth. Despite Ningxiang being a premium local pig breed, the molecular mechanisms underpinning its development and growth are not fully elucidated. To address this, we undertook a comprehensive analysis integrating mRNA-seq, miRNA-seq, and proteomics from Ningxiang pig longissimus dorsi muscles at different developmental stages (30, 90 and 210 days post-birth). This study represents the inaugural integrated analysis of the Ningxiang pig’s longissimus dorsi muscle. To gain deeper insights into proteomics’ functionality, we utilized time-series STEM analysis and devised a network diagram showcasing significant miRNA-mRNA-protein interactions over time.

## 2 Materials and methods

### 2.1 Animals and sampling

Nine pure-bred male Ningxiang pigs were selected for this experiment, categorized into three age groups: 30, 90, and 210 days post-birth, with three samples from each period. Pigs within the three groups are half-siblings, while the repeated samples are from full-siblings. These pigs were sourced from the Ningxiang Pig Farm of Hunan Dalong Animal Husbandry Technology Co., Ltd. They were fed *ad libitum* on a cereal-based commercial diet under standard environmental conditions. All longissimus dorsi muscle samples were collected within 30 min post-slaughter and were promptly snap-frozen in liquid nitrogen, then stored at −80°C until further use. All procedures had three biological replicates. The experiments involving animals were authorized by the Institutional Animal Care and Use Committee of Hunan Agricultural University, Changsha, Hunan Province, China, under approval number 2013-06.

### 2.2 Hematoxylin–Eosin staining and analysis of myofiber size

The longissimus dorsi muscle was removed from the fixation solution, dehydrated using graded alcohols, and then immersed in melted wax at 65°C. Subsequently, the muscle tissue was embedded using an embedding machine. The resultant wax block was sectioned at 4 μm thickness using a paraffin microtome. After dewaxing, sections were air-dried at room temperature. Staining involved hematoxylin for 5 min followed by a water rinse. The slides were then sequentially dehydrated using 85% and 95% alcohol grades and stained with eosin for 5 min. The stained sections were sealed using neutral gum. Under microscopic examination, images were captured and analyzed using Case Viewer 2.4 software. For each sample, three random fields of view were selected and further analyzed using Image-Pro Plus 6.0 software and Graphpad Prism 8.

### 2.3 RNA library preparation, and transcriptome sequencing

First, total RNA is extracted by Trizol reagent (Invitrogen, Life Technologies, Carlsbad, CA, United States) from cells, then small RNA molecules of the target size of 18–30 nt are separated from the total RNA using PAGE electrophoresis. Then, customized linkers are attached to both ends of the small RNA, which contain the sequences needed for subsequent PCR amplification and sequencing. After that, the small RNA is reverse transcribed into cDNA using reverse transcriptase. After obtaining cDNA, it is amplified, and its concentration is detected using Nanodrop 2000 (NanoDrop Technologies, Wilmington, DE, United States) and its integrity is checked using agarose gel electrophoresis, Illumina HiSeq 4000 sequenced the prepared small RNA library. Firstly, Oligo (dT) magnetic beads were used to enrich mRNA and break the enriched mRNA into small fragments suitable for sequencing platform. Reverse transcriptase was used to reverse transcribed the RNA fragments into cDNA. Meanwhile, RNase H was used to remove the RNA, synthesize the second strand of cDNA, form double-stranded cDNA, and repair the end of the cDNA fragment. An “A” base is added to the 3′ end to facilitate subsequent splicing, PCR amplification of the cDNA fragment is performed using primers complementary to the splicing, purification of the PCR product, and quality inspection is performed, including detection of the size, purity, and concentration of the DNA fragment. Finally, the prepared library was deeply sequenced in Shanghai Meiji Biomedical Biotechnology Co., Ltd. (Shanghai, China) (Li et al., 2021).

### 2.4 Transcripts assembly

Using The SeqPrep (https://github.com/jstjohn/SeqPrep) Perform quality control on the data obtained from sequencing using the default parameters in, and then use HISAT2 (https://ccb.jhu.edu/software/hisat2/index.shtml) compare the clean data obtained after quality control with the data from the reference genome (access number PPJNA531381), and then use StringTie to map the data for each sample (https://ccb.jhu.edu/software/stringtie/index.shtml) assemble (Chen et al., 2022).

### 2.5 Alignment and annotation of small RNA

1 μg of total RNA was extracted from tissue samples to start. The TruSeq Small RNA Sample Prep Kits (Illumina, San Diego, CA, United States) were used in this extraction for library construction and reverse transcription. TBS380 (Picogreen) was used for quantitative analysis and sequencing using the Illumina Hiseq 2000/2500 platform. miRDeep2 software was used to predict secondary structures. New miRNAs were identified based on prediction results, considering factors like Dicer restriction site information and energy values.

### 2.6 Prediction of target genes

Use miRanda 3.3a and RNAHybrid software to predict target genes. Please refer to Chen’s articles for specific methods (Chen et al., 2022).

### 2.7 Analysis of DEGs, DEMs and DAPs

Transcripts Per Million (TPM) were used to quantify the gene expression levels. Differential expressed miRNAs and differential expressed mRNAs were pinpointed using DEseq2. Differential abundance proteins were discerned through a *t*-test with *p*-value <0.05. Gene functions were annotated utilizing databases [Nr, ftp (Baumeier et al., 2017)], Protein Family [Pfam (Bhadel et al., 2020)], String (Bogner-Strauss et al., 2010), and [KEGG (Bonkowsky et al., 1979)], respectively.

### 2.8 Time-series expression analysis

STEM was employed to analyze gene expression dynamics across time points. A *p*-value ≤0.05 was deemed significant in the STEM analysis.

### 2.9 miRNA target gene prediction and network analysis

miRNA can regulate the expression of target genes. Therefore, two websites miRanda (http://www.miranda.org/) and RNAhybrid are used to predict the binding sites of miRNA target genes. miRNA-mRNA correlation network was constructed by predicting binding sites and expression correlation using miRanda software. The differentially expressed mRNA was analyzed with GO annotation. Finally, the miRNA-mRNA interaction network was constructed. The constructed interactive network is visualized by cytoscape software.

### 2.10 Mass spectrometry analysis

Q Exactive HF-X mass spectrometer (Thermo, United States) and UltiMate 3000 RSLC nanosystem (Thermo, United States) were used to analyze samples. The specific method is consistent with Li’s article (Li et al., 2022).

### 2.11 Validation of predicted miRNA, mRNA and protein

Total RNA from Ningxiang pig muscle, across 3 stages, was extracted using the Animal Total RNA Kit and subsequently reverse transcribed into cDNA. Primer 5.0 facilitated primer design (refer Supplementary Table S1), with each sample undergoing three reaction iterations. miRNA and mRNA relative expression was calculated using via the comparative CT method (2^–△△CT^). 3 proteins (*APOC3*, *ATP5E*, *PDHA1*) at 3 different time points were used for PRM verification and identification methods refer to the methods in the previous published article (Li et al., 2022).

### 2.12 Statistical analysis

In this study, unpaired two-tailed Student’s t-test was used to measure the level changes of candidate differential mRNA and miRNA at any two different time points. Data were expressed as mean ± SEM.

## 3 Results

### 3.1 Morphological characteristics in muscle of Ningxiang pigs at different stages

**FIGURE 1:**
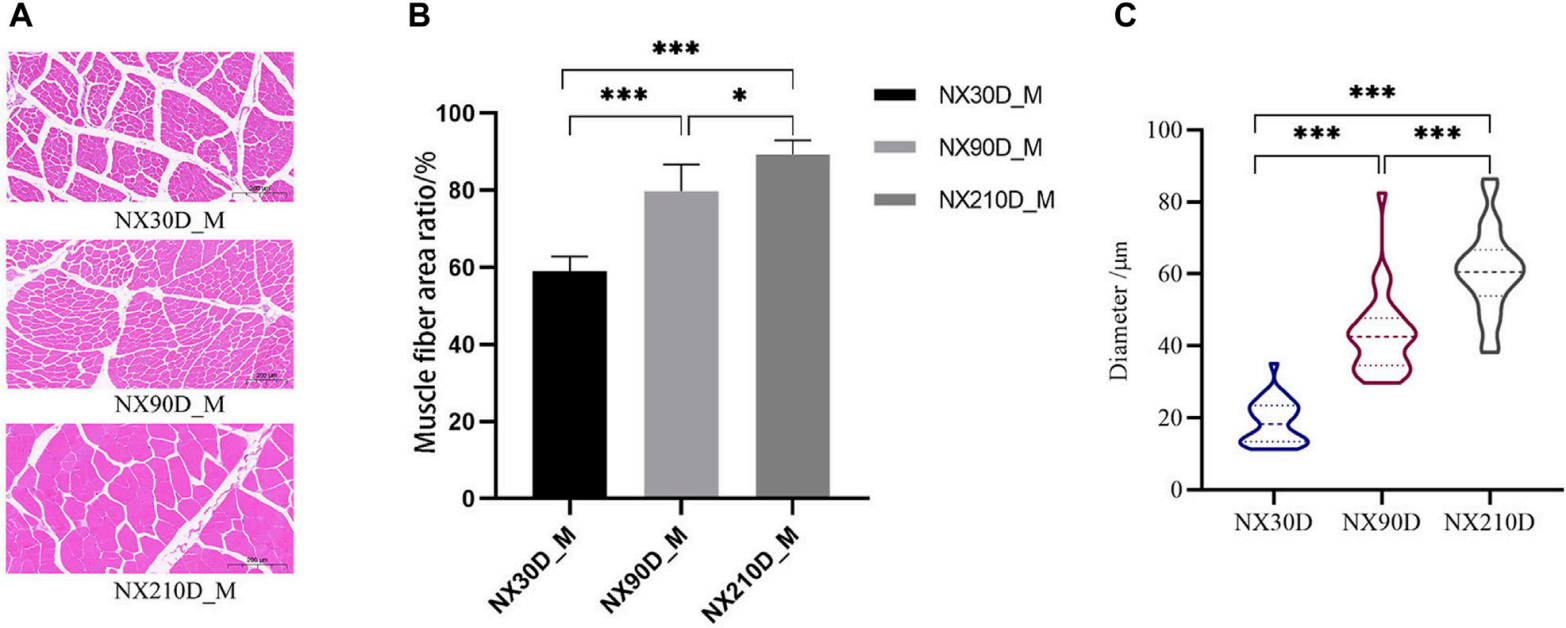
Three different time the H&E staining of muscle, percentage of muscle cells in same scales and the diameter of muscle fibers. **(A)** H&E staining results of Ningxiang pig muscle three developmental stages. **(B)** Percentage of muscle fibers in the longissimus dorsi muscle at 30, 90, and 210 day. **(C)** Average muscle fiber diameter statistics at different developmental stages. *n* = 3, asterisk means *p < 0.05, **p < 0.01, ***p < 0.001.

H&E staining revealed an increase in muscle fibers ratio and muscle diameter in muscle tissue at 90 and 210 days when compared to 30 days (Figure 1). These observations suggest that postnatal muscle tissue in Ningxiang pigs undergoes several biological processes. Consequently, a comprehensive study was conducted on the biological significance of miRNA, mRNA, and protein during Ningxiang pigs’ developmental phase using multi-omics sequencing analysis.

### 3.2 Identification of transcriptome, miRNA omics and proteome

**FIGURE 2:**
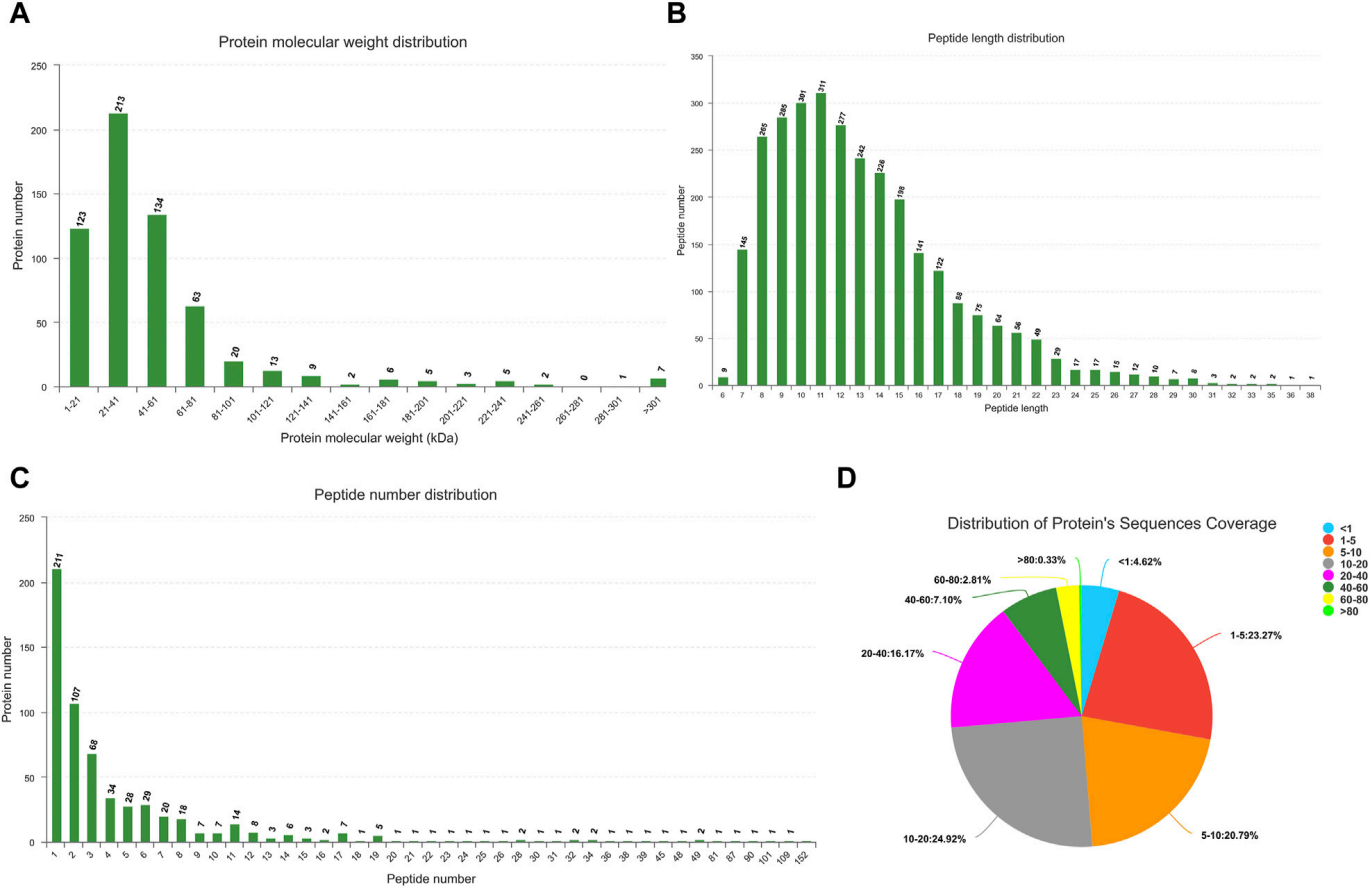
Characterization of proteins in Longissimus dorsi muscle. **(A)** Protein molecular weight distribution. **(B)** Distribution of peptide length. **(C)** Quantitative distribution of peptides. **(D)** Distribution of protein’s sequences coverage.

An analysis identified 19,653 known mRNAs and 2,758 novel mRNAs. Additionally, comprising 427 known miRNAs and 739 novel ones were discerned (Supplementary Files S1–S3). Utilizing the Proteome Discoverer software for processing MS/MS spectra, 376,823 spectra were detected in Ningxiang pig muscle tissue protein, identifying 2,980 peptides (Supplementary Figure S1). Figure 2 presents the characterization of proteins in Longissimus dorsi muscle of Ningxiang pig.

### 3.3 Analysis of differentially Expressed proteins

**FIGURE 3:**
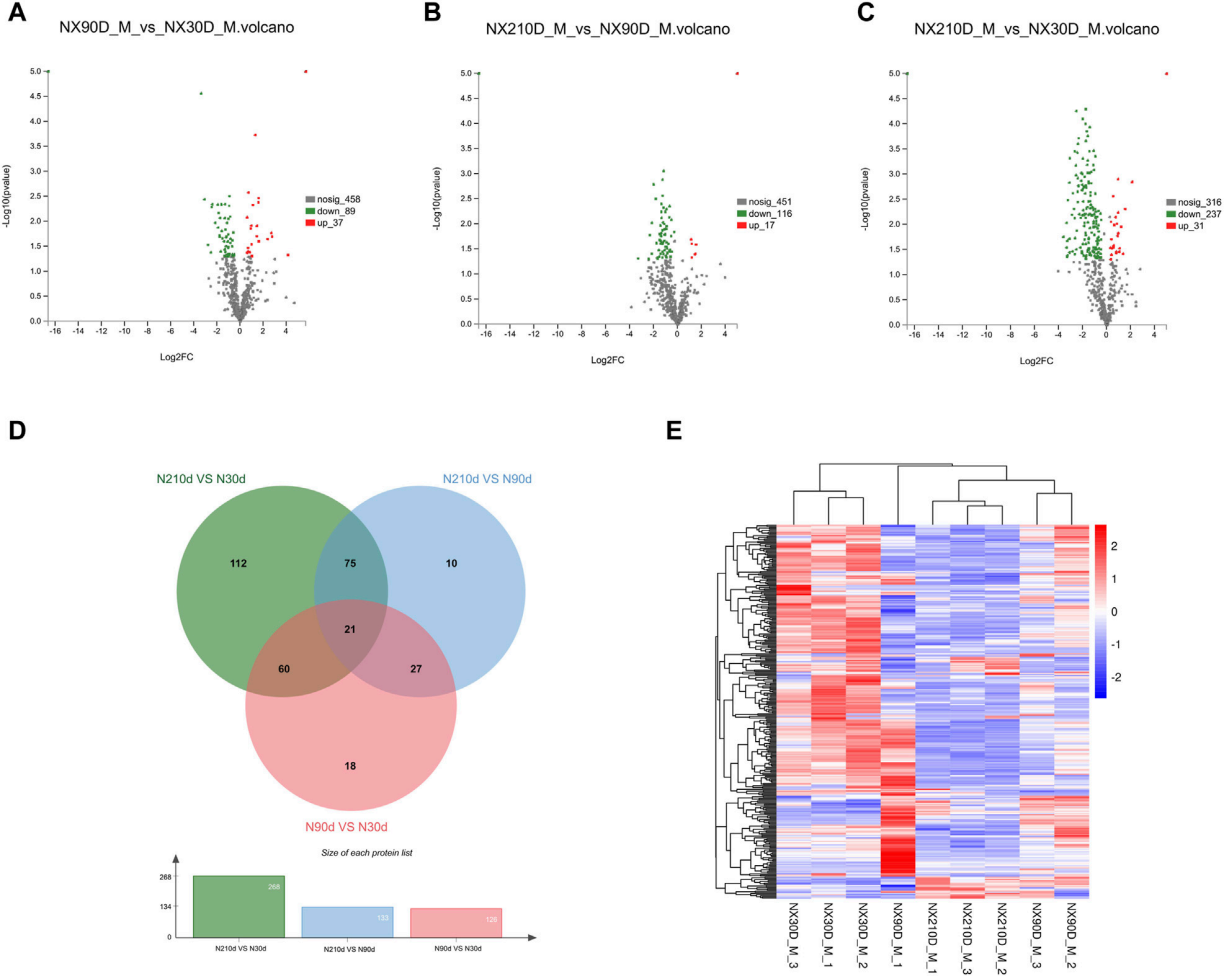
Profiling of DAPs. **(A–C)** Variations in protein expression in Longissimus dorsi muscle. **(D)** Venn diagram illustrating DAPs across three comparative groups. **(E)** Protein expression levels at 30, 90, and 210 days.

The results highlighted 572 differentially expressed proteins among the three groups (Figures 3A–C): NX90D_M vs. NX30D_M identified 126 DAPs, with 89 downregulated and 37 upregulated. The NX90D_M vs. NX210D_M comparison revealed 133 DAPs, with 116 being downregulated and 17 upregulated. For NX210D_M vs. NX30D_M, 268 DAPs were identified, 237 downregulated, and 21 upregulated. Data analysis indicated that NX210D_M vs. NX30D_M had the highest number of DAPs. Moreover, unique differential expressions were found in the groups NX90D_M vs. NX30D_M (18 DAPs), NX210D_M vs. NX90D_M (10 DAPs), and NX210D_M vs. NX30D_M (112 DAPs), while 21 DAPs being co-expressed across all groups (Figure 3D, Supplementary File S4). Hierarchical clustering was employed to analyze the protein expression of DAPs in Ningxiang pig muscle across three distinct periods (Figure 3E).

**FIGURE 4:**
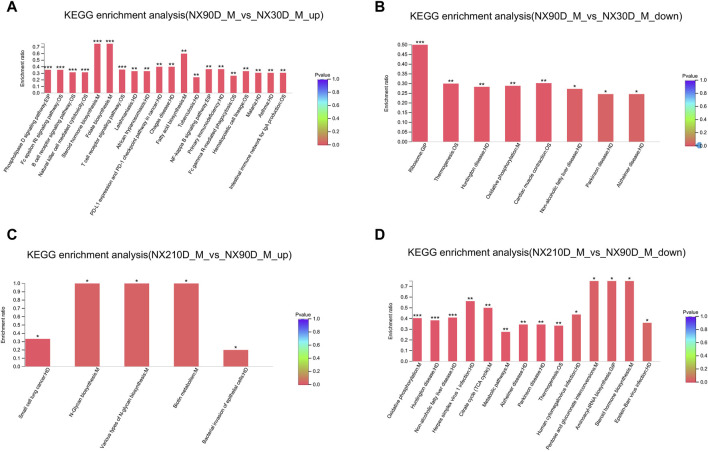
KEGG analysis of DAPs for the NX90D_M vs. NX30D_M and NX210D_M vs. NX90D_M groups. **(A,B)** Upregulated and downregulated KEGG pathways for DAPs in the NX90D_M vs. NX30D_M comparison. **(C,D)** Upregulated and downregulated pathways for DE proteins in the NX210D_M vs. NX90D_M comparison. Rank the top 20 enriched KEGG pathways based on their *p*-values, with significance represented by *p < 0.05, **p < 0.01, ***p < 0.001.

To gain deeper insights into the DAPs across the three Ningxiang pig muscle stages, KEGG enrichment was utilized to analyze adjacent DAP time points. Upregulated DAPs in the NX90D_M vs. NX30D_M comparison were predominantly enriched in 20 pathways, surpassing the eight pathways for downregulated DAPs (Figures 4A, B, Supplementary Files S5, S6). Pathways like Steroid hormone biosynthesis, Folate biosynthesis, Phospholipase D signaling, and Fatty acid biosynthesis, which are integral to muscle growth and differentiation, were found to be significantly enriched. Furthermore, in the NX210D_M vs. NX90D_M comparison, upregulated DAPs were enriched in four pathways, while downregulated DAPs appeared in 14 pathways (Figures 4C, D, Supplementary Files S7, S8). These findings underscore the significance of the period from 30 to 90 days in Ningxiang pig muscle development.

### 3.4 Combined proteome and transcriptome analysis in muscle tissue

**FIGURE 5:**
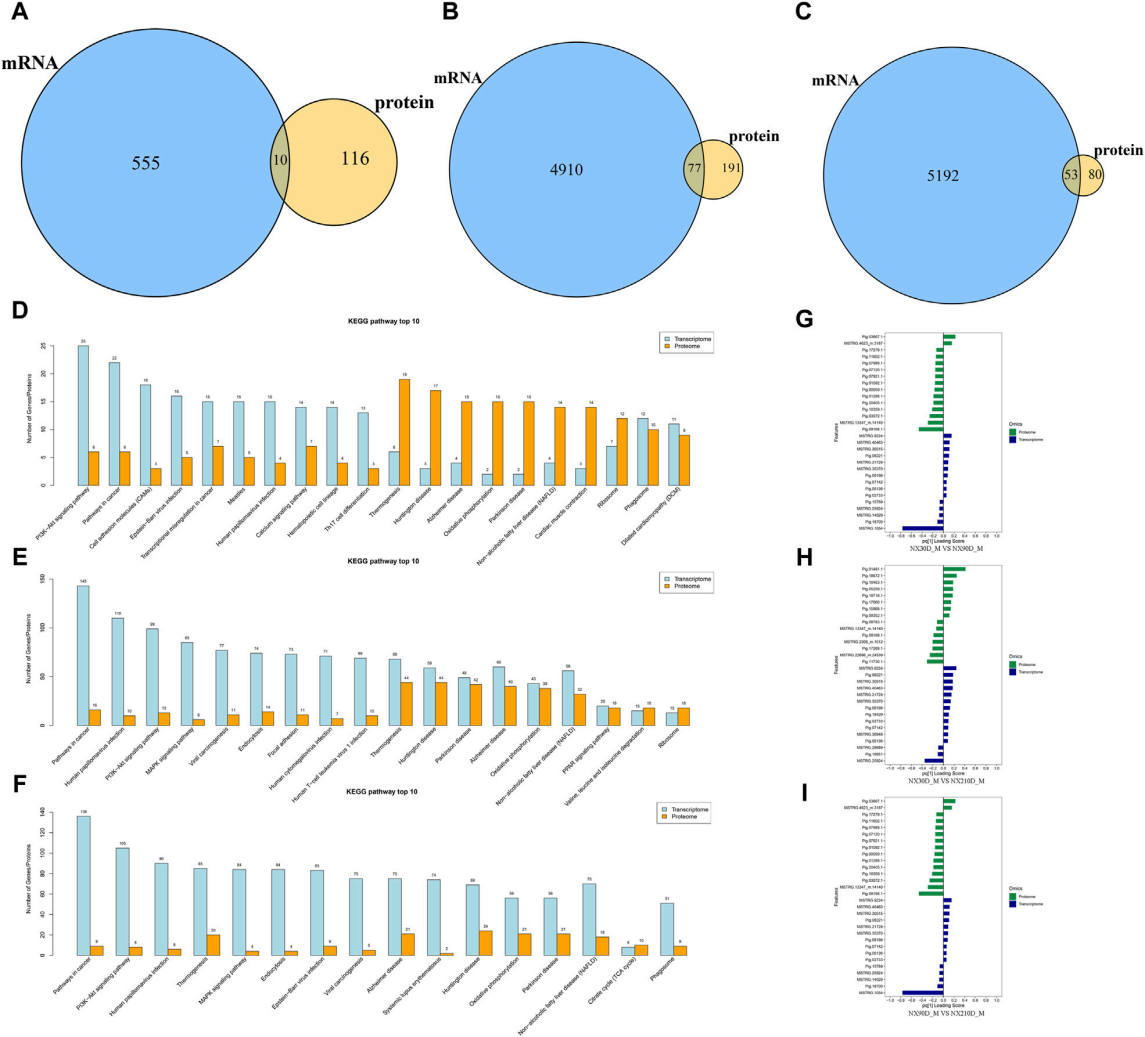
Overlap between DAPs and DEGs. **(A)** Analysis of associated DAPs and DEGs in NX90D_M vs. NX30D_M. **(B)** Analysis of associated DAPs and DEGs in NX210D_M vs. NX30D_M. **(C)** Analysis of associated DAPs and DEGs in NX210D_M vs. NX90D_M. **(D)** Top 10 KEGG pathways for DAPs and DEGs (NX90D_M vs. NX30D_M). **(E)** Top 10 KEGG pathways for DAPs and DEGs (NX210D_M vs. NX30D_M). **(F)** Top 10 KEGG pathways for DAPs and DEGs (NX210D_M vs. NX90D_M). **(G)** Two-way Orthogonal Partial Least Squares (O2PLS) analysis to intuitively evaluate the correlation between datasets for NX90D_M vs. NX30D_M. **(H)** O2PLS analysis to evaluate the correlation between proteome and transcriptome datasets for NX210D_M vs. NX30D_M. **(I)** O2PLS analysis to evaluate the correlation between datasets for NX210D_M vs. NX90D_M.

To investigate the association betweenDAPs and differentially expressed genes (DEGs), the transcriptome and proteome of the respective groups were juxtaposed and scrutinized. For the NX90D_M vs. NX30D_M comparison, 565 DEGs were identified, with 10 mRNA/proteins found to be expressed at this stage. For the NX210D_M vs. NX30D_M comparison, 4,987 DEGs were identified, with 268 showing co-expression. Similarly, in the NX210D_M vs. NX90D_M comparison, 5,245 DEGs were identified, with 133 demonstrating co-expression (refer to Figures 5A–C and Supplementary Files S9–S11). The pathway enrichment across the three timeframes differs from that observed in proteomics.

To elucidate the relationship between genes and proteins within each group, KEGG pathway enrichment analysis was employed. The results revealed a significant overlap between DAPs and DEGs (see Supplementary Figures S2–S4), with the majority focused on metabolism, immunity, protein synthesis, and degradation (as shown in Figures 5D–F). This implies that these DAPs and DEGs critically influence key pathways during the muscle development of Ningxiang pigs.

The Two-way Orthogonal Partial Least Squares (O2PLS) method was utilized to intuitively ascertain the correlation between the two datasets. By computing the loading values for each protein and gene, and assessing the absolute values of these loadings across both datasets, the top 15 proteins and genes were pinpointed (see Figures 5G–I and Supplementary Files S12–S14). Those with high loading values are deemed pivotal for the congruence of the two datasets. The figures suggest that the loading value variances for the top 15 are most pronounced in the NX210D_M vs. NX30D_M comparison and least pronounced in the 90-day vs. 30-day comparison. This leads to the inference that the concordance between the transcriptome and proteome of Ningxiang pigs likely augments as they age.

### 3.5 Combined analysis of miRNA, transcriptome and proteomics

**FIGURE 6:**
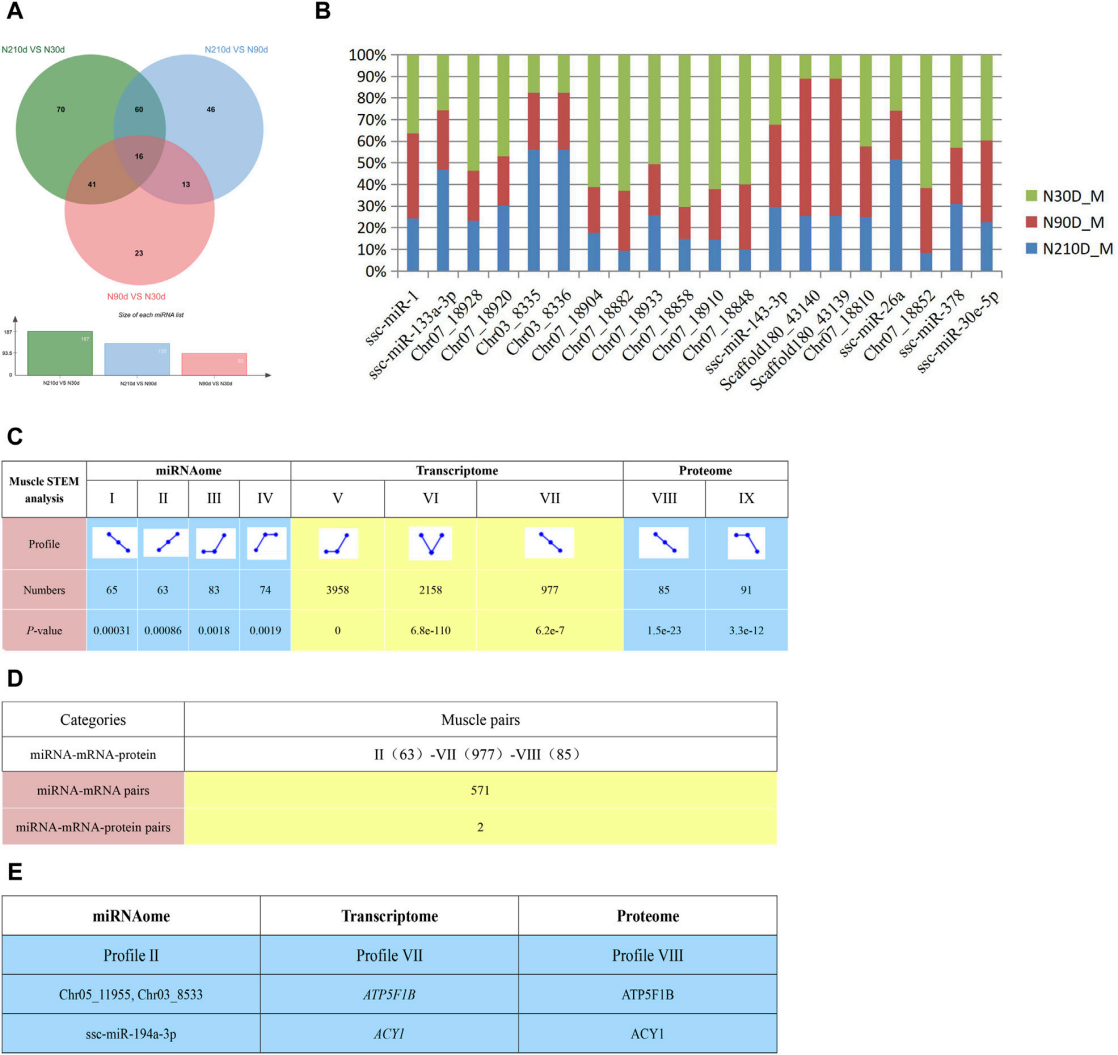
Potential negative interactions among 3 omics. **(A)** DEMs across three comparison groups. **(B)** Top 20 miRNAs expressed in the Longissimus dorsi muscle. **(C)** STEM analysis detailing miRNA, transcriptome, and proteome interactions, including the count and associated *p*-value for miRNA, mRNA and protein in each profile. **(D)** Enumeration of potential negative interactions of miRNA–mRNA and 3 omics based on STEM expression patterns. **(E)** Two potential interaction pairs from STEM analysis. STEM analysis uses clustering algorithm to trend all genes and proteins of three omics at three different time points, and then genes or proteins with the same trend will be classified into one module, and Ⅱ, Ⅶ and Ⅷ are the names of these corresponding modules.

Comparing the three stages, 415 DEMs were identified. Specifically, 23, 70, and 46 DEMs were distinctively expressed in NX90D_M vs. NX30D_M, NX210D_M vs. NX90D_M, and NX210D_M vs. NX30D_M comparisons, respectively, with 16 DEMs co-expressed across all groups (Figure 6A). Notably, certain highly expressed miRNAs are linked to muscle development (e.g., ssc-miR-1, ssc-miR-133a-3p, ssc-miR-378) and fatty acid metabolism (e.g., ssc-miR-143, ssc-miR-26a, ssc-miR-30e) (Figure 6B).

To elucidate the interplay of miRNA, transcriptomics, and proteomics across the Ningxiang pig muscle tissue’s developmental stages, we employed the STEM analysis which be used to cluster, compare, and visualize gene expression data from short time series, identifying important temporal expression profiles and the genes associated with these profiles. Results indicate significant enrichment in certain expression profiles across the stages (Figure 6C, *p* < 0.05), with dynamic expression observed during muscle development. This identified 571 negative miRNA-mRNA interactions (Figure 6D and Supplementary File S15) spanning Profile II (63) to Profile VII (977). Integrating proteomic data from STEM analysis revealed two negative miRNA-mRNA-protein interactions (Figure 6E).

### 3.6 RT-qPCR quantification of miRNAs and mRNAs

**FIGURE 7:**
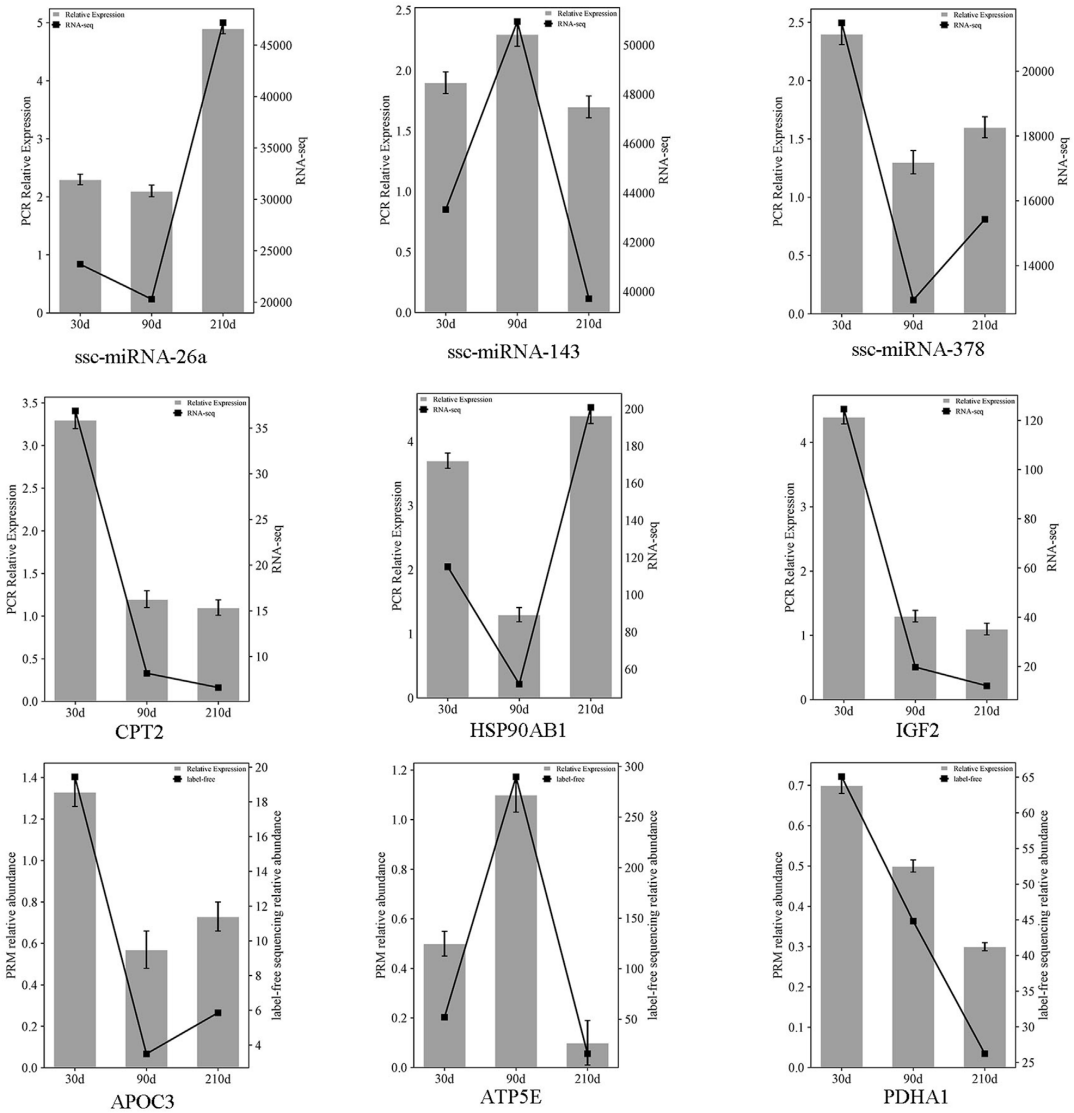
Comparative expression of three selected miRNAs, mRNAs, and proteins across three stages intervals, referencing miRNAome/transcriptome and qRT-PCR, and proteome with PRM. The double Y-axis diagram illustrates the results of qRT-PCR/PRM validation (with label-free sequencing relative abundance quantified in millions). Data are presented as mean ± SEM, with *n* = 3 per group.

Three miRNAs (ssc-miR-378, ssc-miR-143, ssc-miR-26a), mRNAs (*CPT2*, *IGF2*, *HSP90AB1*), and proteins (*APOC3*, *ATP5E*, *PDHA1*) were chosen at random and quantified using RT-qPCR and PRM across three developmental stages (30, 90 and 210 days post-birth). The results exclude false positives in sequencing data (Figure 7).

## 4 Discussion

During the growth and developmental stages of organisms, intricate molecular regulations constantly occur *in vivo*, predominantly governed by protein receptors, transcription factors, and exocrine secretions. The intensity of these adjustments varies with life’s progression. Pigs, significant to global agriculture due to their rapid growth, substantial yield, and good meat quality, have gained prominence. The incorporation of Duroc, Landrace, and Large White breeds in China has indeed augmented meat availability. However, as China progresses towards affluence, consumer preferences for pork’s taste and flavor have evolved. Research indicates that local pig breeds generally offer superior pork texture and intramuscular fat (IMF) content compared to European breeds. According to the Ningxiang Municipal Government, a total of 446,100 Ningxiang pigs were sold in 2022, achieving a total industrial chain output value of 4,193 billion yuan. This study conducted in-depth research on the muscle tissue of Ningxiang pigs at three developmental stages using proteomics and transcriptomics.

In our analysis, we identified 572 DAPs in the Ningxiang pig’s longissimus dorsi muscle across different comparisons: NX90D_M vs. NX30D_M, NX210D_M vs. NX90D_M, and NX210D_M vs. NX30D_M. KEGG pathway enrichment revealed that upregulated DAPs in the NX90_M vs. NX30D_M comparison predominantly participate in growth (e.g., Steroid hormone biosynthesis) and immunity pathways. Conversely, downregulated DAPs are primarily linked to physiological maintenance functions. This suggests that between 30 and 90 days of development, DAPs crucially influence Ningxiang pig development and immunity enhancement while also playing a regulatory role in physiological sustenance. In the early growth phase, due to the organ’s incomplete development and cell differentiation, protein expression tends to be elevated compared to the subsequent stages. However, for the NX210D_M vs. NX90D_M comparison, upregulated DAPs chiefly partake in metabolic processes such as glucose utilization and protein metabolism such as T-complex protein-1 (*TCP1*) is a ubiquitous group II chaperonin and is known to fold various proteins, such as actin and tubulin. meantime *LdTCP1γ* interacts with all subunits of *TCP1* complex as well as other proteins belonging to pathways like metabolic process, ribosome, protein folding, sorting, and degradation (Yadav et al., 2022). Concurrently, downregulated DAPs emphasize immune-related pathways. This underlines that as pigs mature, their physiological systems become more refined, and DAPs increasingly contribute to immune and inflammatory functions.

In this result (Figure 3D in Supplementary File S4), we identified 21 co-expressed DAPs (such as *APOC3*, *NDUFA2*, *HSPD1*, *ATP5E*, *PDHA1*) across three times intervals. Apolipoprotein C-III (*APOC3*) primarily characterizes very low-density lipoprotein (*VLDL*) cholesterol but is also present in high-density lipoprotein (*HDL*) and low-density lipoprotein (*LDL*) cholesterol. As a structural protein in *CM*, *VLDL*, and *HDL, APOC3* is pivotal in the apolipoprotein C family, chiefly inhibiting lipoprotein lipase activity and the liver’s uptake of triacylglycerol-rich lipoproteins and residues (Akoumianakis et al., 2021). The *PDHA1* gene encodes the E1 alpha protein, which, when combined with the E1 beta protein from the *PDHB* gene, constitutes the E1 enzyme. This enzyme is part of the pyruvate dehydrogenase complex, essential for transforming food-derived energy into a cellularly useable form (Imbard et al., 2011). Studies have indicated that *PDH* inhibition augments dependence on extracellular lipids, revealing a growth defect in certain cell lines which can be reversed by supplying exogenous free fatty acids (Rajagopalan et al., 2015). These co-expressed DAPs may be integral to muscle growth and IMF deposition.

mRNA serves as a critical conduit in transmitting genetic information for protein synthesis. However, the relationship between DEGs and DAPs remains elusive. In this investigation, high-throughput sequencing was employed to juxtapose DEGs and DAPs in Ningxiang pigs from lactation to fattening, marking the inaugural comprehensive analysis of these pigs’ DEGs and DAPs. We identified 19,653 known mRNAs and 2,758 novel mRNAs in developing muscle. Remarkably, 74 DEGs were co-expressed across all three stages, with 13 newly predicted mRNAs (e.g., *HPN-204*) and 61 recognized mRNAs (like *PLIN3*, *CPT2*) being overexpressed. For instance, Perilipin 3, a Perilipins protein family member, it mainly targets newborn lipid droplets (LDs) and plays a regulatory role in the biogenesis and presentation of lipid droplets. In skeletal muscle cells, the presence of PLIN3 contributes to the formation and maintenance of lipid droplets, especially during lipolysis, where PLIN3 affects the breakdown of lipids and the release of energy by interacting with lipolysis enzymes (Lee et al., 2018). Carnitine palmitoyl transferase-2 (*CPT2*) is an important enzyme that plays a role in regulating fatty acid metabolism in muscle cells. *CPT2* is responsible for binding long-chain fatty acids to carnitine, allowing them to enter the mitochondria for oxidative metabolism to produce energy. *CPT2* activity can affect the extent to which muscle cells can utilize fatty acids, which in turn affects muscle energy supply and metabolism. The energy generated by the metabolic process of long chain fatty acids can not only meet the basic metabolic needs of muscles, but also provide energy support for muscle synthesis. Therefore, *CPT2* has a critical effect on muscle growth and repair ability. Overall, *CPT2* affects muscle energy supply and growth by regulating fatty acid metabolism and energy supply (Rolver et al., 2023). Fatty acids are a major source of energy for the heart and muscles. These co-expressed DEGs suggested that the high intramuscular fat content of Ningxiang pigs might be related to these high expression DEGs.

In this study, 16 DEMs were co-expressed across three stages. These DEMs include 7 unidentified miRNAs (e.g., Chr07_18848, Chr07_18852) and 9 known ones such as ssc-miR-10383 and ssc-miR-299. Notably, miR-299-5p significantly reduced *ATF2* mRNA and protein levels in A549 cells (Gao et al., 2020), and *ATF* proteins are implicated in embryonic myogenesis mediated by Wnt signals (Chen et al., 2005), indicating a potential role for the ssc-miR-299-*ATF2*-Wnt axis in muscle progenitor cells. Silva et al. (2019) demonstrated that miR-29c binds to *MuRF1*’s 3′UTR to mediate repression. Overexpression of miR-29c in the tibialis anterior led to a 40% increase in muscle mass, augmented fiber cross-sectional area and force, a 30% enhancement in length, and boosted satellite cell proliferation and differentiation. In C2C12 cells, miR-29c oligonucleotides increased differentiation levels, as shown by heightened *eMHC* immunostaining and the myotube fusion index. Concurrently, the mRNA levels of myogenic markers rose. An analysis of DEMs revealed numerous DEMs, including ssc-miR-1 (Schiffer et al., 2021), ssc-miR-133a (Wang et al., 2021), ssc-miR-378 (Podkalicka et al., 2022) et al., associated with fat deposition and muscle development. Such findings underscore the pivotal role these miRNAs play in muscle development and fat deposition in Ningxiang pigs.

STEM analysis indicated that protein-coding genes and miRNAs are dynamically expressed during muscle development, often exhibiting inverse patterns. As miRNAs typically suppress their target genes, it is conjectured that miRNA expression often inversely correlates with miRNA-target gene expression in dynamic analyses. For instance, miRNA-mRNA pairs, such as ssc-miR-29a-3p-*PLIN3*, associated with lipid anabolism and cell proliferation, align with the negative regulation observed in STEM analysis. The STEM analysis identified two regulatory networks of miRNA-mRNA-Protein, including Chr05_11955-*ATP5F1B*-ATP5F1B and ssc-miR-194a-3p-*ACY1*-ACY1. Muscle growth depend on intracellular protein synthesis and metabolism and the *ATP5F1B* protein is involved in regulating the level of ATP in cells, which is one of the energy sources required for protein synthesis and metabolism. Therefore, the *ATP5F1B* protein plays an important role in the growth of muscle cells (Ganetzky et al., 2022). *ACY1*, the most prevalent aminoacylase, participates in the hydrolysis of N-acetylated proteins, with significant hydropotonia observed in *ACY1*-deficient children (Sass et al., 2006). These insights emphasize that Ningxiang pig muscle tissue development involves complex regulation, setting the stage for future molecular studies on muscle and fat control.

## 5 Conclusion

The current investigation delineates the expression profiles and functional networks of miRNAs, mRNAs, and proteins in the Longissimus dorsi muscle of Ningxiang pigs across three developmental phases. A total of 572 differentially expressed DAPs were identified, predominantly exerting a negative regulatory influence. Among these, we detected 21 co-expressed DAPs, 74 co-expressed DEGs, and 16 co-expressed DEMs specific to the growth and developmental stages of Ningxiang pigs. KEGG enrichment analysis indicated that the majority of these co-expressed entities are associated with muscle growth and IMF deposition. Through STEM correlation analysis, 571 miRNA-mRNA pairs were recognized, and two specific miRNA-mRNA-protein pairs were discerned across the three omics. These findings provide foundational knowledge for future studies on miRNA-mRNA-protein interactions in Ningxiang pigs and grant novel perspectives on tri-omic functions in the Longissimus dorsi muscle throughout its developmental timeline.

## Data Availability

The datasets presented in this study can be found in online repositories. The names of the repository/repositories and accession number(s) can be found in the article/Supplementary Material.
