# Supplementary figures and images for: Integrated analysis of muscle transcriptome, miRNA, and proteome of Chinese indigenous breed Ningxiang pig in three developmental stages
Source: Front Genet. 2024 May 14;15:1393834. doi: 10.3389/fgene.2024.1393834 (PMC11130441; doi:10.3389/fgene.2024.1393834)

# Protein information

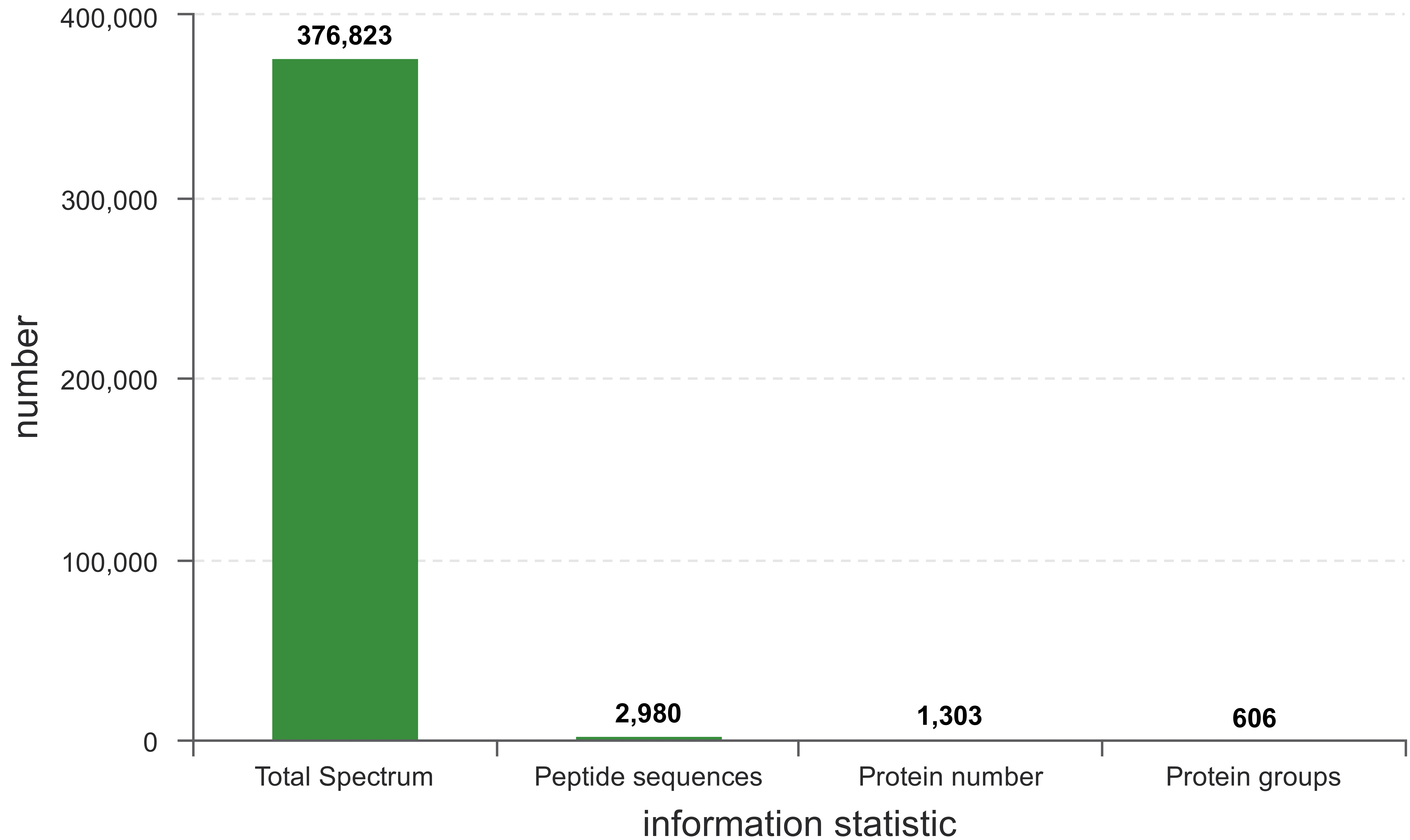

Supplement: Supplementary file 1 [file DataSheet1.ZIP › Supplementary figure-1 protein-infomation.pdf]

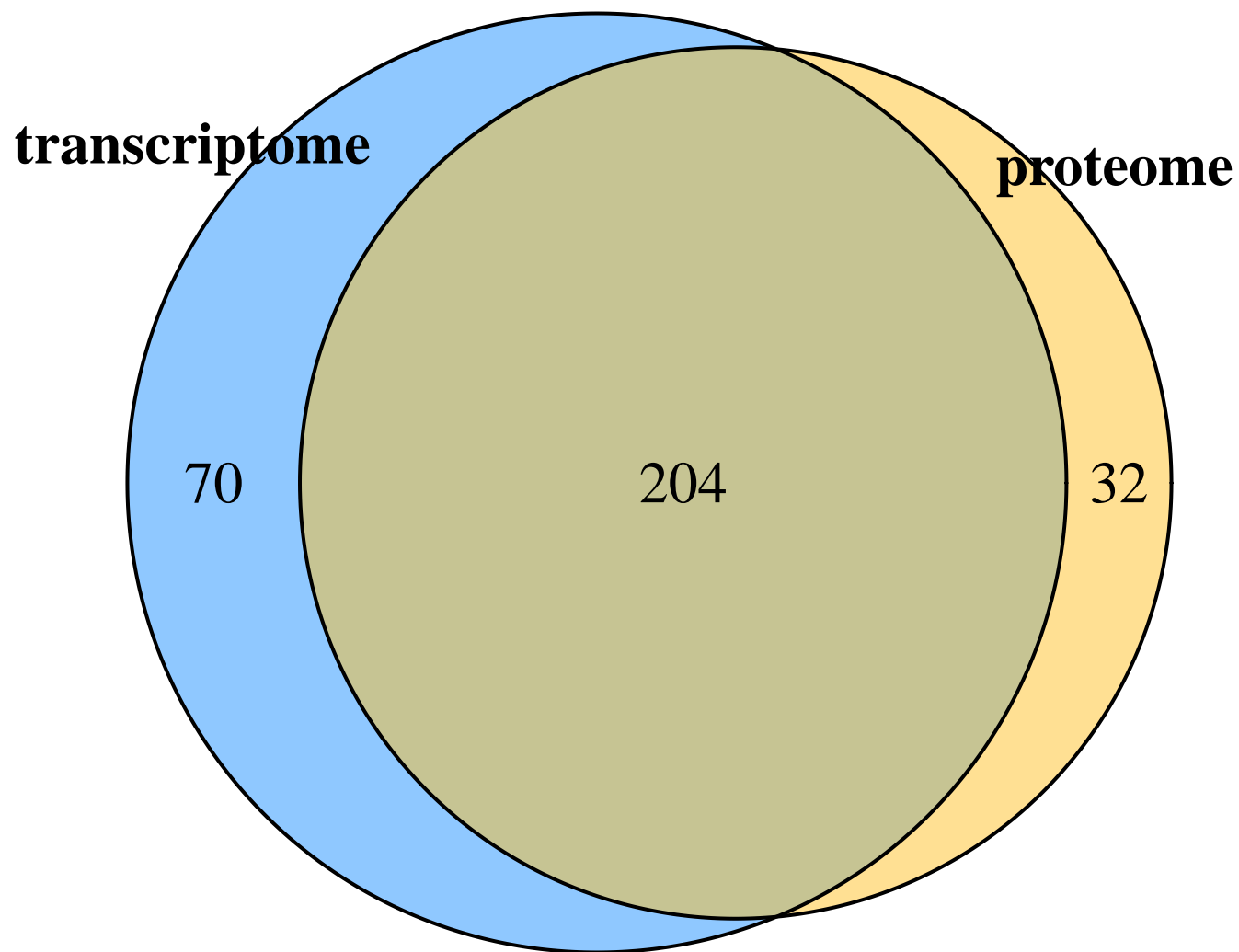

Supplement: Supplementary file 1 [file DataSheet1.ZIP › Supplementary figure-2 NX90D_M_vs_NX30D_M.paths.Venn.pdf]

**transcriptome**

**proteome**

65

257

17

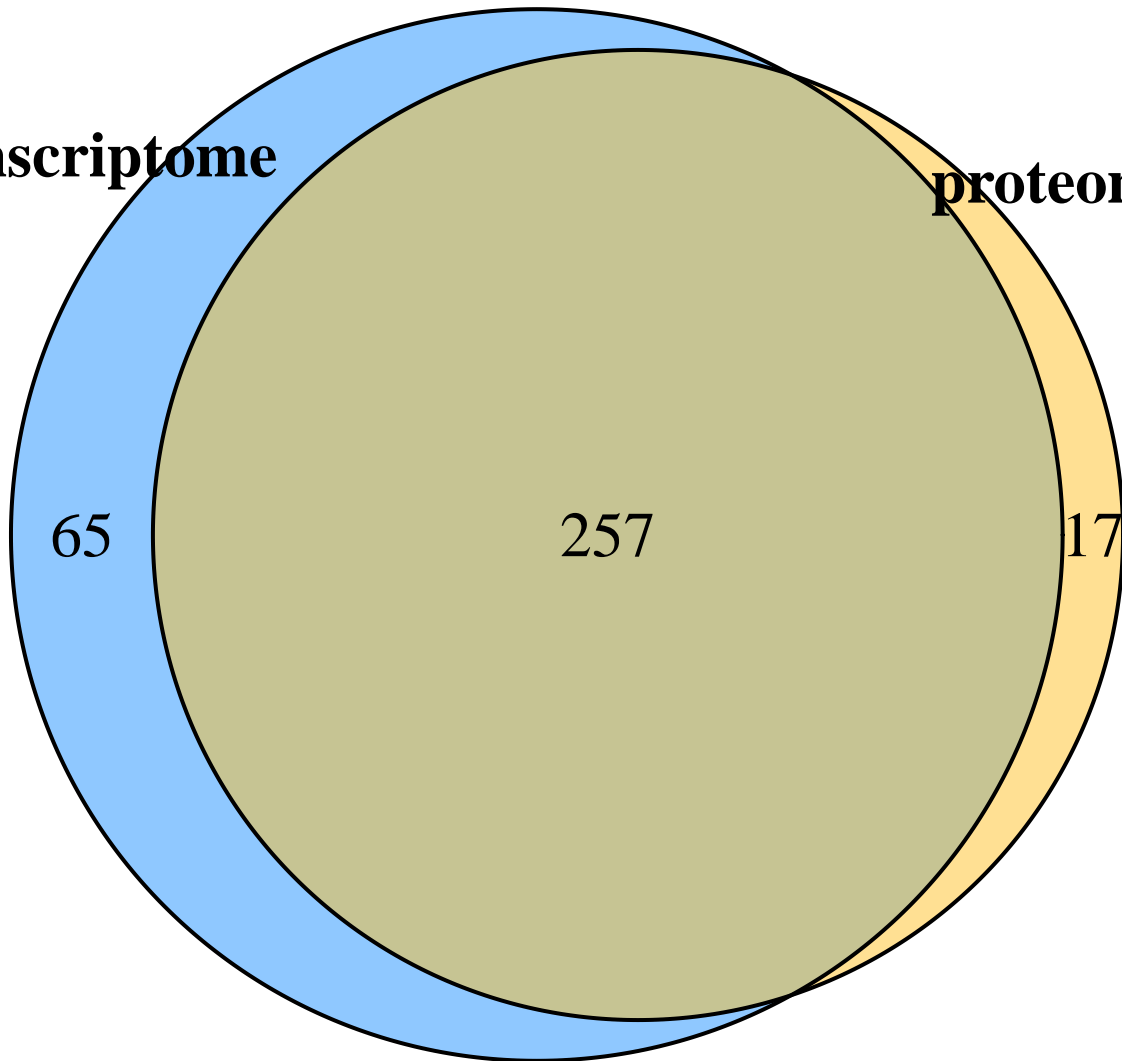

Supplement: Supplementary file 1 [file DataSheet1.ZIP › Supplementary figure-3 NX210D_M_vs_NX30D_M.paths.Venn.pdf]

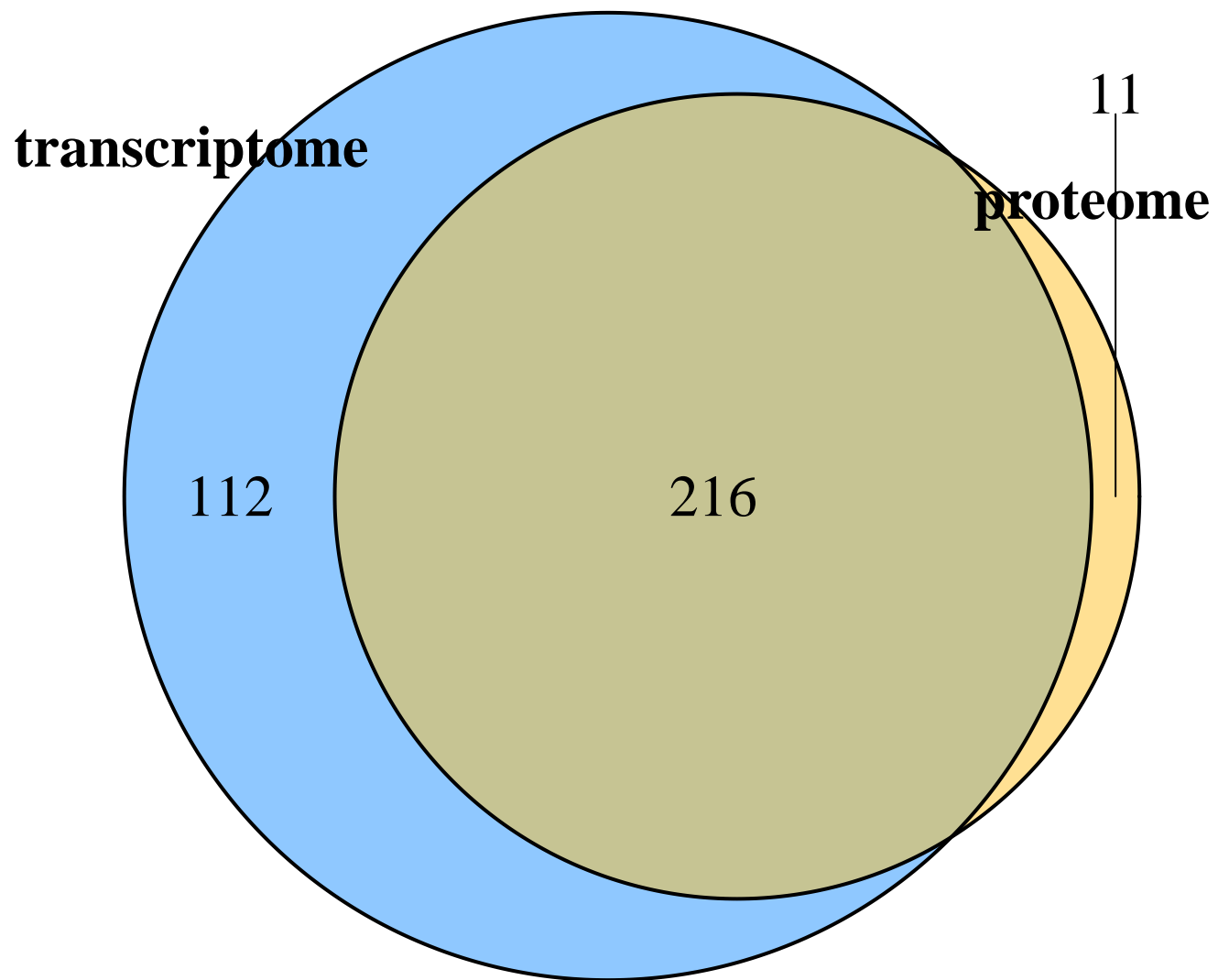

Supplement: Supplementary file 1 [file DataSheet1.ZIP › Supplementary figure-4 NX210D_M_vs_NX90D_M.paths.Venn.pdf]
