# Supplementary material for: Integrated analysis of muscle transcriptome, miRNA, and proteome of Chinese indigenous breed Ningxiang pig in three developmental stages
Source: Front Genet. 2024 May 14;15:1393834. doi: 10.3389/fgene.2024.1393834 (PMC11130441; doi:10.3389/fgene.2024.1393834)
Supplement: Supplementary file 1 [file DataSheet1.ZIP › Supplementary Table 1 Primers sequence.doc]

| Ssc-miR-378 | F : CTCCTGACTCCAGGTCCTGT |
| --- | --- |
|  | R : GCCTTCTGACTCCAAGTCCA |
| Ssc-miR-143 | 5’-GCGTGAGATGAAGCACTGTAGCTC-3’ |
| Ssc-miR-26a | 5’-TTCAAGTAATCCAGGATAGGCT-3’ |
| CPT2 | F: CAAGGCCTACCCTCTGGATA |
|  | R: GCTCACAATCTTCCCGTCTT |
| IGF2 | F: GTGGCATCGTGGAAGAGTGC |
|  | R: CCAGGTGTCATAGCGGAAGAA |
| HSP90AB1 | F: TGGAGAGTTCTACAAAAGCCTG |
|  | R: CTTCAAAATCTTGCTCTGCTG |

Supplementary Table 1

The primers used for qRT-PCR in this study.
